# 

**Published:** 1841-01-23

**Authors:** 


### BOOKS RECEIVED.

*Stokes on the Chest.* Dublin edition. *From the author.* This work was left in the Custom House from some mistake, and only reached us recently. We return our acknowledgments to the author at a late period.

(We are requested to mention that Messrs. Haswell & Barrington have for sale a few copies of the Dublin edition.)

*Stokes' Lectures on Practice*, edited by Dr. Bell, with additional lectures. A notice of this work will appear in our next. (*From the Publishers.*)

*Louis on Typhoid Fever.* Second edition' considerably enlarged. (*From the author.*) A notice will appear at an early date.

*A System of Practical Medicine*, 3d vol., by Alexander Tweedie, M. D. *Diseases of the Organs of Respiration*, with notes and additions by W. W. Gerhard, M. D. (*From the publishers.*)

